# Structural and Parametric Optimization of S–CO_2_ Nuclear Power Plants

**DOI:** 10.3390/e23081079

**Published:** 2021-08-19

**Authors:** Nikolay Rogalev, Andrey Rogalev, Vladimir Kindra, Ivan Komarov, Olga Zlyvko

**Affiliations:** 1 Department of Thermal Power Plants, National Research University “Moscow Power Engineering Institute”, 111250 Moscow, Russia; rogalevnd@mpei.ru; 2 Department of Innovative Technologies of High-Tech Industries, National Research University “Moscow Power Engineering Institute”, 111250 Moscow, Russia; rogalevan@mpei.ru (A.R.); komarovii@mpei.ru (I.K.); zlyvkoov@mpei.ru (O.Z.)

**Keywords:** supercritical carbon dioxide, thermodynamic cycle, power plant, optimization, efficiency

## Abstract

The transition to the use of supercritical carbon dioxide as a working fluid for power generation units will significantly reduce the equipment′s overall dimensions while increasing fuel efficiency and environmental safety. Structural and parametric optimization of S–CO_2_ nuclear power plants was carried out to ensure the maximum efficiency of electricity production. Based on the results of mathematical modeling, it was found that the transition to a carbon dioxide working fluid for the nuclear power plant with the BREST–OD–300 reactor leads to an increase of efficiency from 39.8 to 43.1%. Nuclear power plant transition from the Rankine water cycle to the carbon dioxide Brayton cycle with recompression is reasonable at a working fluid temperature above 455 °C due to the carbon dioxide cycle′s more effective regeneration system.

## 1. Introduction

### 1.1. Promising Way to Increase Efficiency and Decrease the Capital Cost of Nuclear Power Plants

Today in the Russian Federation, there are 11 nuclear power plants (NPPs) with 38 operating power units and a total installed capacity of 30.3 GW. The majority of them, 55%, are equipped with a pressurized water reactor (PWR), and the most widely used among them is the VVER–1000 type. Besides the PWRs, NPPs also operate fast breeder reactor (FBR) BN–800 with sodium heat carrier, 2100 MW heat power, 800 MW supplied power, and 39.4% net efficiency. Moreover, a new reactor facility BREST–OD–300 is under development. This FBR has a lead heat carrier, a two–circuit heat transport to the steam turbine and supercritical steam parameters. A common feature of these NPPs is the presence of the steam turbine cycle, which determines the mass and dimensions of the main energy equipment, especially of steam turbines, and rather low thermal efficiency.

A prospective method for increasing the NPP fuel efficiency and its investment reduction is an application of the supercritical carbon dioxide (S–CO_2_) working fluid. It allows for use of the Brayton cycle with low auxiliary power consumption, moderate initial temperature, and compact dimensions of the main energy equipment [1,2,3]. This direction has been actively developed since the middle of the past century [4,5,6,7]. This area of development arises scientists′ interests around the world largely because of carbon dioxide′s competitive advantages compared to other working fluids.

**Figure 1:**
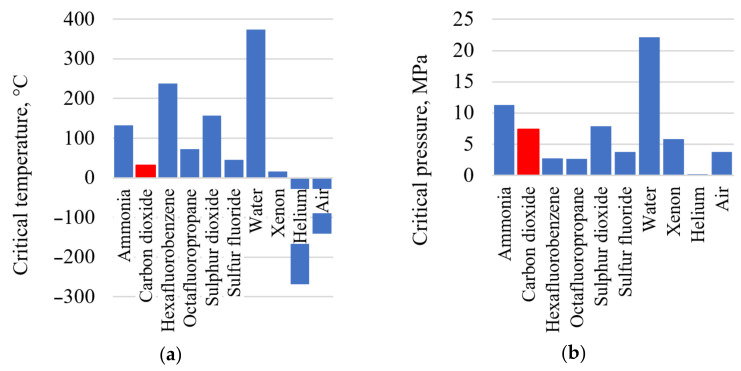
Thermophysical performance of the promising heat carriers: (**a**) heat carrier critical temperature; (**b**) heat carrier critical pressure.

The carbon dioxide heat carrier has a low critical temperature of 30.98 °C and a pressure of 7.38 MPa (Figure 1). The low carbon dioxide critical temperature is near to the ambient atmosphere, which allows the working fluid compression near the saturation line (the CO_2_ density is quite high at the near–critical parameters) [8,9], reducing the compressor work and the temperature of heat removed from the cycle without the working fluid condensing. Besides, carbon dioxide is less aggressive than water and shows its corrosion activity only in the presence of moisture content in the working fluid or with a water film on a metal surface. The carbon dioxide and water working fluid prices are compatible.

The advantages of carbon dioxide compared to air and water in the role of the working fluid are caused by its thermophysical performance (Table 1) in conditions typical for steam and gas turbine facilities. The high density of carbon dioxide at given turbine inlet and outlet conditions leads to S–CO_2_ turbine mass and dimensions being smaller than those of the steam or gas turbines. The airfoil grid friction losses in an S–CO_2_ turbine will be smaller than those in a steam or gas turbine because of carbon dioxide′s smaller viscosity [10].

### 1.2. A State-of-the-Art Review of the S–CO_2_ Brayton Cycles

**Figure 2:**
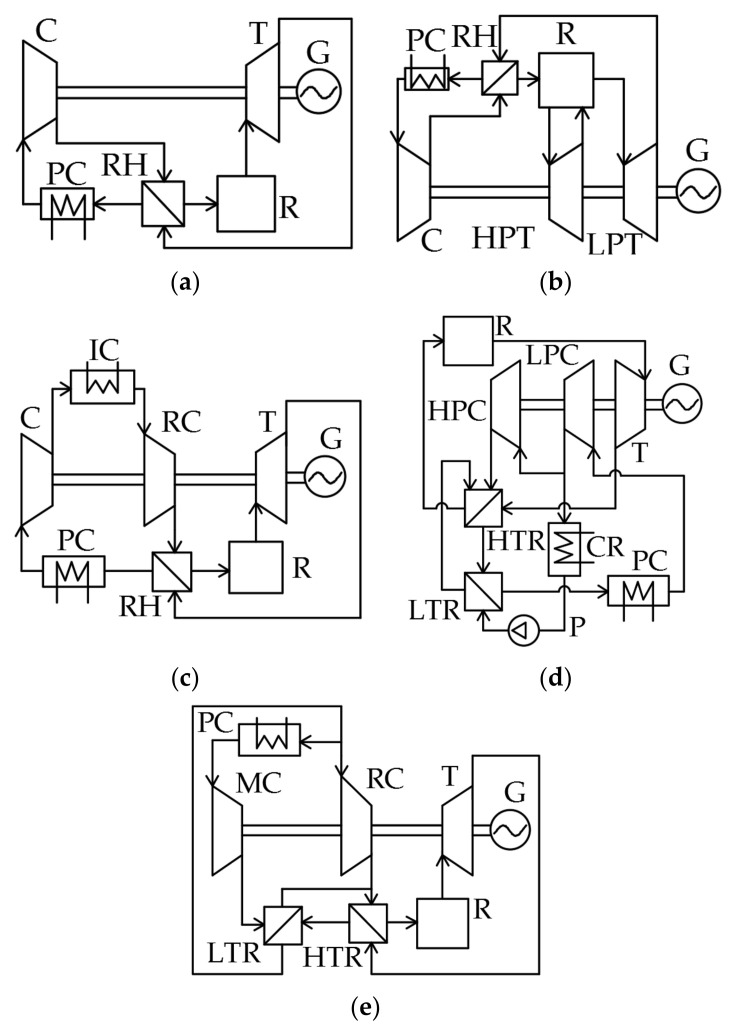
Supercritical CO_2_ Brayton cycles: (**a**) S–CO_2_ Brayton cycle with regeneration; (**b**) S–CO_2_ Brayton cycle with reheating; (**c**) S–CO_2_ Brayton cycle with intermediate cooling; (**d**) S–CO_2_ Brayton cycle with partial cooling.; (**e**) S–CO_2_ Brayton cycle with recompression.

Long-term thermodynamic studies of S–CO_2_ power facilities resulted in the development of the five cycles presented in Figure 2.

The simplest S–CO_2_ cycle is a closed Brayton cycle with the heat utilization of the exhaust gases (Figure 2a). The recirculating carbon dioxide enters the compressor (C), then the compressed fluid enters the regenerator (RH). The pre-heated flow enters the reactor (R) where its temperature increases. Then, the supercritical fluid is sent to the turbine (T) that drives the electricity generator (G). After the expansion, the turbine exhaust gas enters the regenerator, where it transfers heat to the compressed working fluid. Before it enters the compressor, the cooled CO_2_ flow is sent to the cold source, or pre-cooler (PC), where the working fluid is additionally cooled.

The thermal efficiency is about 42% for 550 °C initial temperature, 32 °C compressor inlet temperature, and 25 MPa compressor outlet pressure. Studies of the turbine inlet pressure′s influence upon the S–CO_2_ Brayton cycle′s efficiency show that its increase by 2 MPa in the range of 13.8–27.6 MPa causes an efficiency increase of 0.71% [11], and a 150 °C initial temperature increase leads to a thermal efficiency increase of 5.5%.

Reheating (Figure 2b) may increase the S–CO_2_ Brayton cycle efficiency. This cycle differs from the previous one (Figure 2a) by the second working fluid supply to the reactor. Pressurized carbon dioxide flow is sent by the compressor to the regenerator, where it is heated by the low-pressure turbine (LPT) outlet flow. Then, the flow is sent to the reactor for its temperature increase up to the cycle initial temperature. After that, the flow is sent to the high-pressure turbine (HPT) flow path, where it expands to half of the initial pressure. Then, the flow re-enters the reactor for reheat. Afterward, the CO_2_ flow expands in the LPT and enters the regenerator, where it transmits heat to the compressor outlet flow. From the regenerator outlet, the hot flow is sent to the cooler, and then it re-enters the compressor.

The introduction of the working fluid reheat is followed by an increase of turbine outlet temperature, which increases the reactor inlet temperature and the mean integral temperature of heat supply to the cycle. Thermodynamic studies [7,12] reveal that the efficiency increase is maximal when the high-pressure turbine expansion ratio is equal to the low-pressure turbine one. The cycle efficiency is about 41.5% at 550 °C and 25 MPa initial cycle parameters and the turbine outlet pressure of 7.5 MPa. However, it should be noted that the introduction of an additional superheater will inevitably cause pressure losses in it. A 10% increase in pressure losses in the range of 0–250 kPa reduces the facility efficiency by 0.11%. With a further increase of the superheater pressure losses, application of the reheat will not be reasonable because it reduces the cycle efficiency. The introduction of the secondary reheat allows the supercritical CO_2_ Brayton cycle efficiency increase by 0.20 to 0.26% at the heater pressure losses below 125 kPa.

One more method for the efficiency increase of the simplest supercritical CO_2_ Brayton cycle is the introduction of intermediate cooling that reduces the compressor energy consumption (Figure 2c). In contrast to the regeneration cycle (Figure 2a), this cycle uses low- pressure compressor (LPC) and high-pressure compressor (HPC) as well as an intermediate cooler (IC). After the low-pressure compressor, the working fluid passes the cooler, where it cools and enters the high-pressure compressor, where it reaches the cycle initial parameters. The maximal increase of this cycle′s thermodynamic efficiency is reached when the second compressor pressure ratio is 1.5–1.9 times higher than the first compressor one. In this case, the S–CO_2_ Brayton cycle efficiency increases by 0.8%.

In addition to the above schemes, one more way toward S–CO_2_ Brayton cycle efficiency improvement is the use of partial cooling (Figure 2d) [6]. This cycle differs from the cycle with regeneration by the application of a high-temperature regenerator (HTR) and low-temperature regenerator (LTR), condenser (CR), pump (P), and recompressing compressor (RC). The flow pressurized in the compressor is split into two parts: the first is sent to the RC and the second is cooled in the condenser and also compressed by the pump. The second flow of compressed CO_2_ is heated in the LTR by the exhaust gas heat and then mixes with the first CO_2_ flow in the HTR. After that, the flow is supplied to the reactor, where it is heated up to the cycle′s initial temperature. The reactor exhaust supercritical CO_2_ enters the turbine that drives the power generator. Then, the expanded gas is directed to the sequentially connected HTR and LTR for heat utilization. Afterwards, the flow is cooled in the pre-cooler before it enters the compressor. This solution improves the regeneration system efficiency by means of the heat exchangers′ operation at different pressures. The S–CO_2_ Brayton cycle has 44.8% efficiency at a 550 °C cycle initial temperature and 25 MPa initial pressure.

Figure 2e shows the supercritical CO_2_ Brayton cycle with recompression, which is a simplified modification of the partial cooling cycle presented in Figure 2d. This cycle differs from the partial cooling cycle by the absence of a pump and condenser. The carbon dioxide flow is split into two parts upstream of the pre-cooler. The first part is cooled and supplied to the main compressor, where it reaches the initial parameters. The second part is directly supplied to the recompressing compressor, where it is also compressed up to the initial cycle pressure. After the main compressor, the compressed CO_2_ flow enters the LTR, where it is heated by the outlet carbon dioxide flow up to the temperature equal to the second CO_2_ flow temperature after the RC. Then, the two flows merge and enter the HTR, where they are also heated up to the reactor inlet temperature by heat utilization of the exhaust gases. Then, the reactor heats the working fluid up to the cycle′s initial temperature. Then, the flow enters the turbine that drives the power generator. After the flow expands in the turbine, the hot exhaust gases are cooled in the sequentially connected HTR and LTR and then split into two flows again.

This scheme solves the problem of low efficiency of the exhaust gases′ heat utilization. This problem is related to the remarkable difference of the regenerator hot and cold flows′ specific heat capacity. This difference is a specific feature of the S–CO_2_ Brayton cycle with regeneration. In other words, the split of compressed flow and introduction of the LTR and HTR provides deeper utilization of the exhaust gases heat and smaller heat losses in the cooler. The result of this technical solution is that the S–CO_2_ Brayton cycle efficiency increases up to 46%.

### 1.3. Summary of the Thermodynamic Investigation Results for the S–CO_2_ Brayton Cycles

Most of the published thermodynamic investigations are obtained at incomparable conditions, at different input data and calculation methods. As a result, one can see different evaluations of similar factors′ influence on thermal efficiency. For example, the study of the S–CO_2_ recompression Brayton cycle efficiency [11] shows that a 100 °C increase in the turbine inlet temperature in the 500–1000 °C range increases the efficiency by a 2.0% average, but according to the results in [6], this increase is 3.3%. On the other side, study [7] shows that a 100 °C increase of the turbine inlet temperature in the 550–850 °C range increases the mean cycle efficiency by 4%, and according to [13], in the 550–700 °C range, the same temperature increase leads to the 5% increase in efficiency.

Table 2 summarizes the main results of thermodynamic studies [6,7,11,13,14] of the S–CO_2_ recompression Brayton cycle. The results show a remarkable evaluation difference and determine the relevance of the comparison of carbon dioxide cycles under comparable conditions.

To ensure the possibility of an objective comparison of the thermodynamic efficiency of S–CO_2_ power cycles, a preliminary optimization of key parameters was carried out using the Aspen Plus software package. Table 3 summarizes the main thermodynamic performance of the five cycles described above. The supercritical CO_2_ Brayton cycle with recompression has a maximal net efficiency of 47.28% at the initial temperature and pressure of 550 °C and 35 MPa. The high working fluid temperature at the heat supply source inlet of the S–CO_2_ recompression Brayton cycle defines its prospects for the NPP reactor heat utilization.

Despite numerous studies′ results on the CO_2_ operating NPPs, many questions are not yet answered. Specifically, the studies do not consider the nuclear reactor influence upon the outer CO_2_ circuit [15,16]. Therefore, this work is devoted to the optimization of thermal flow parameters in NPPs, including actual operation conditions and limits for three nuclear reactors including VVER-1000, BN-800, and BREST-OD-300.

## 2. Research Object

### 2.1. Existing Nuclear Reactors and Operation Regimes′ Limitations

The VVER-1000 reactor is a vertical cylindric vessel with an elliptical floor. Inside the vessel are placed the active zone and the internal elements. The reactor has two circuits. In the first circuit, the non-boiling water circulates with around 16 MPa pressure.

The heat carrier pressure in the first circuit is limited by the reactor vessel material strength performance. Water enters the reactor at 289 °C temperature and is heated up to 322 °C. Then, it travels along with four circulation loops, or “hot” runs, to the steam generator where it transmits its heat to the second circuit heat carrier. The main circulation pumps return the water to the reactor by “cold” strings. The 322 °C reactor outlet temperature is limited with the 347.4 °C vaporization temperature at 16 MPa. In the case of vaporization, the boiling crisis occurs in the active zone, which results in a sudden rapid increase of the fuel elements′ wall temperatures, which crucially shortens their lifetime.

The second circuit working fluid is H_2_O, which produces power. The turbine inlet is supplied with 1633 kg/s of primary steam at a 274 °C temperature and 5.9 MPa pressure. The feed water temperature at the steam generator inlet is about 220 °C. This unit efficiency is about 31.7%. In changing the steam circuit to the carbon dioxide one, the possibility must be retained for the first circuit′s water cooling from 322 down to 289 °C.

Besides the PWRs, NPPs operate the FBR BN-800 with sodium heat carrier. This reactor has 2100 MWt thermal power, 800 MWe supply power, and 39.4% net efficiency. The sodium heat carrier shortcoming is its disruptive reaction with water that produces water vapor. The water ingress in the reactor degrades the active zone′s cooling and accelerates the structural material corrosion.

The reactor′s first circuit sodium outlet temperature is limited not by the heat carrier saturation temperature, but by the fuel element cell temperature limit. The fuel element shell is made of the austenitic steel with a 650 °C temperature limit. The first circuit outlet temperature is only 547 °C because of the fuel element thermal non-uniformity, the cold sodium leakage past the active zone possibility, and the active zone outlet sodium temperature non-uniformity. In turn, the 354 °C reactor entry sodium temperature is limited by strength characteristics. In particular, the reduction of the active zone′s heat carrier temperature would require its velocity increase, which may cause equipment vibration.

The Na-Na heat exchangers located in the reactor vessel transfer heat from the first circuit to the second one. In the intermediate circuit, the sodium temperature difference is 180 to 200 °C. In this reactor, the second circuit heat carrier at the Na-Na heat exchanger entry and outlet temperatures are 309 °C and 505 °C, respectively. The intermediate circuit pressure is 1.8 MPa higher than the first circuit one to prevent radioactive sodium leakage.

The third circuit heat carrier is H_2_O, which produces the work. The turbine inlet conditions are 875 kg/s primary steam mass flow, 485 °C temperature, and 14.2 MPa pressure. The feed water temperature at the steam generator inlet is about 210 °C.

The change of the third circuit heat carrier from water to carbon dioxide may improve the NPP efficiency. Papers [17,18] show that at the moderate 450–700 °C turbine inlet temperature, S–CO_2_ cycles might be more efficient than the traditional Rankine cycle.

Although CO_2_ does not actively react with sodium with the production of a lot of vapor and heat, in this case the second circuit is still needed. As mentioned before, the Na-Na heat exchangers are located inside the reactor vessel, so this technical solution retains the reactor structure; only the steam equipment and the steam generator shall be changed. The retained second circuit sodium temperature at the Na-Na heat exchanger inlet and outlet will provide the reactor stability.

The new FBR facility with lead heat carrier twin circuit heat supply to the turbine and supercritical steam parameters BREST-OD-300 is being developed now. This reactor′s advantages are the heat carrier′s natural circulation and a capability of more complete use of uranium fuel. One more important factor is the natural safety application. In the crash case, the pool full of lead, where the reactor is located, will release excessive heat into the atmosphere.

The BREST-OD-300 reactor thermal and electric power values are 700 MWt and 300 MWe, respectively. In the first reactor, the circuit circulates the liquid lead with the active zone inlet and outlet temperatures of 420 °C and 535 °C, respectively. In the steam generator, the hot liquid metal transfers its heat to the second circuit. In the steam generator, water heats up to 505 °C at 17 MPa pressure, then it enters the turbine, expands, produces work, and enters the condenser. The condensate passes the regeneration system heaters, where it is heated up to 340 °C. Then, the feed water pump sends it back to the steam generator.

The application of the second circuit carbon dioxide cycle may require its structure to change and its dimensions to increase. The steam generators are located in the concrete pool together with the reactor but not in the reactor vessel. This allows retainment of the BREST-OD-300 initial design.

Table 4 summarizes the main performance of three reactors used for the NPP computer simulation. The hot flow temperature at the steam generator inlet for the BREST-OD-300, BN-800, and VVER-1000 reactors may be 535 °C, 505 °C, and 322 °C, respectively. According to the analysis [6], the higher the cycle initial temperature is, the larger the difference is between the traditional Rankine and the supercritical Brayton cycles. Therefore, at the initial analysis of the reactor performance, it is possible to assume that the working fluid change from water to carbon dioxide will result in the largest efficiency increase in the BREST-OD-300 reactor.

### 2.2. Schemes and Parameters for the Promising S–CO_2_ Nuclear Power Plants

**Figure 3:**
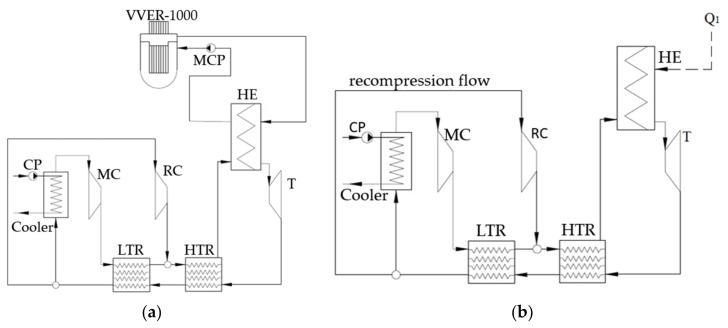
Schemes of the S–CO_2_ nuclear power plants: (**a**) VVER-1000 NPP reactor; (**b**) BN-800/ BREST-OD-300 NPP reactor.

Figure 3a presents a heat flow chart of the closed carbon dioxide cycle with the VVER-1000 nuclear reactor and recompression; below, its principle is described. The water heat carrier leaves the reactor at 322 °C temperature. Then, the flow travels to the surface twin-flow heat exchanger (HE), where it cools down to 289 °C and transmits its energy to the carbon dioxide circuit. The carbon dioxide flow is heated in the heat exchanger up to 315 °C at 20 MPa pressure. Then, it expands in the carbon dioxide turbine (T) to 7.6 MPa.

Then, the working fluid enters the sequentially connected high-temperature and low-temperature regenerators, where the turbine exhaust gas transfers its heat to the CO_2_ pressurized in the compressors. The gas cooled in the regenerators is split into two flows, the main and recompression. The recompression gas is 29% of the total mass flow. It is sent to the recompressing compressor. The main flow is sent to the cooler, where its temperature drops down to 32 °C, which improves the main compressor efficiency. In the cooler, the cooling agent is water at 1.3 bar and 15 °C supplied by the circulation pump. After the main flow is compressed up to the 20 MPa supercritical pressure, it is heated in the low-temperature regenerator. The remaining flow part passes by the cooler and enters the recompressing compressor where its pressure grows up to 20 MPa. The recompressing compressor outlet flow merges with the main flow heated in the low-temperature regenerator. Then, it is heated in the high-temperature regenerator and sent to the heat exchanger that transfers heat to the carbon dioxide cycle. Thus, the cycle is closed.

Concepts of the NPP carbon dioxide outer circuits with the BN-800 and BREST-OD-300 reactors are similar. Figure 3b shows the circuit heat flow chart. In this figure, line Q_1_ shows the heat supply from the reactor to the outer circuit. In this case, the difference with the VVER reactor scheme is the higher initial temperature, 505 °C for the BN-800 and 535 °C for the BREST-OD-300 reactors, and absence of main circulation pump (MCP) because of natural circulation.

Table 5 summarizes the NPP thermal analysis input data. The optimization calculations assumed the carbon dioxide cycle minimal pressure as 7.4 MPa and a minimal temperature of 32 °C.

## 3. Methods

Aspen Plus is the computer program for power production facilities′ computer simulation. The working fluid thermophysical parameters are taken from the NIST Reference Fluid Thermodynamic and Transport Properties Database (REFPROP) high accuracy database [19,20]. The method for thermodynamic processes calculation is briefly explained below.

During the simulation of the NPP heat flow scheme, the turbine inlet temperature was assumed within the limits of the reactor heat carriers. The carbon dioxide circuit mass flow was determined out of the reactor thermal power (ref. Table 4) and the assumed turbine inlet temperature.

At the end of expansion in the turbine, the enthalpy may be calculated as the following:

(1) $$h_{outlet.\mathrm{CO}2-T}=h_{inlet.\mathrm{CO}2-T}-\left(h_{inlet.\mathrm{CO}2-T}-h_{outlet.is.\mathrm{CO}2-T}\right)\cdot \eta_{\mathrm{CO}2-T}$$

where $h_{outlet.\mathrm{CO}2-T}$, $\;h_{inlet.\mathrm{CO}2-T}$—working fluid enthalpy at the turbine outlet and inlet, kJ/kg; $h_{outlet.is.\mathrm{CO}2-T}$—working fluid enthalpy at the turbine outlet at isentropic expansion, kJ /kg; $\eta_{\mathrm{CO}2-T}$—turbine isentropic efficiency.

At the end of the compression process in the compressor, enthalpy is calculated as:

(2) $$h_{outlet.C}=h_{inlet.C}+\left(h_{outlet.is.C}-h_{inlet.C}\right)/\eta_{C},$$

$h_{outlet.C}$, $h_{inlet.C}$—working fluid enthalpy at the compressor outlet and inlet, kJ/kg;
$h_{outlet.is.C}$—working fluid enthalpy at the compressor outlet at isentropic expansion, kJ/kg;
$\eta_{C}$—compressor isentropic efficiency.

The carbon dioxide compressor consumes minimal power when the compression goes near the carbon dioxide subcritical area [21]. Therefore, the turbine′s final expansion pressure is assumed as 7.5 MPa at the minimal cycle temperature of 32 °C. In optimization, the pressure lower limit is assumed as 7.4 MPa to retain the compression in the supercritical area.

Mass flow rates of the cold streams entering the LTR and HTR are equal to the mass flow rates leaving the MC and RC, respectively, which depend on the recompressing ratio. The high- and low-temperature heat exchangers simulation assumed a minimal temperature difference of 5 °C [22].

The circulation pump power is calculated based on cooling water pressure and mass flow.

The optimization criteria is the facility net efficiency, calculated with the following equation:

(3) $$\eta_{net}^{\mathrm{NPP}}=\frac{N_{\mathrm{CO}2-T}\cdot \eta_{mech}\cdot \eta_{eg}-(N_{MC}+N_{RC}+N_{CP}+N_{MCP})/\cdot \eta_{mech}\cdot \eta_{em}}{Q_{t}}\cdot \eta_{tr}\cdot \eta_{r}\cdot \eta_{hx}\cdot {\eta_{hx}}^{\prime },$$

$N_{\mathrm{CO}2-T}$—turbine internal power, MW;
$N_{MC}$—main compressor internal power, MW;
$N_{RC}$—recompressing compressor internal power, MW;
$N_{CP}$—circulation pump internal power MW;
$N_{MCP}$—reactor main circulation pump internal power (to be involved in forced circulation), MW;
$Q_{t}$—reactor thermal power, MW;
$\eta_{mech}$—mechanical efficiency, %;
$\eta_{eg}$—power generator efficiency, %;
$\eta_{em}$—electric motor efficiency, %;
$\eta_{tr}$—heat transportation efficiency, %;
$\eta_{hx}$—heat exchanger inter-channel efficiency, %;
${\eta_{hx}}^{\prime }$—heat exchanger inter-channel efficiency (applied for the heat carrier two circuit reactor), %;
$\eta_{r}$—nuclear reactor efficiency, %.

The following parameters are assumed as the main variables influencing the carbon dioxide NPP efficiency:

- Turbine inlet pressure *p*_0_, MPa;
- Turbine exhaust pressure *p_ex_*, MPa;
- Recompression ratio *x*, %.

The parametric optimization method was the sequential enumeration. First, the turbine inlet pressure *p*_0_ values were varied with 2 MPa step. The investigated variable ranges for the reactors VVER-1000, BN-800, and BREST-OD-300 were 12–32, 12–40, and 19–31 MPa, respectively. The next stage was a variation of the turbine outlet pressure *p*_c_ with 0.5 MPa step for reactors VVER-1000, BN-800, and BREST-OD-300 in ranges 7.4–12, 7.4–13, and 7.5–10.5 MPa, respectively. At the optimal pressure values, the recompression rate *x* was varied with the 5% step for reactors VVER-1000, BN-800, and BREST-OD-300 in ranges 10–50%, 10–50%, and 20–50%, respectively.

## 4. Results

### 4.1. Thermodynamic Optimization of the Turbine Inlet and Outlet Pressure for the S–CO_2_ NPPs

**Figure 4:**
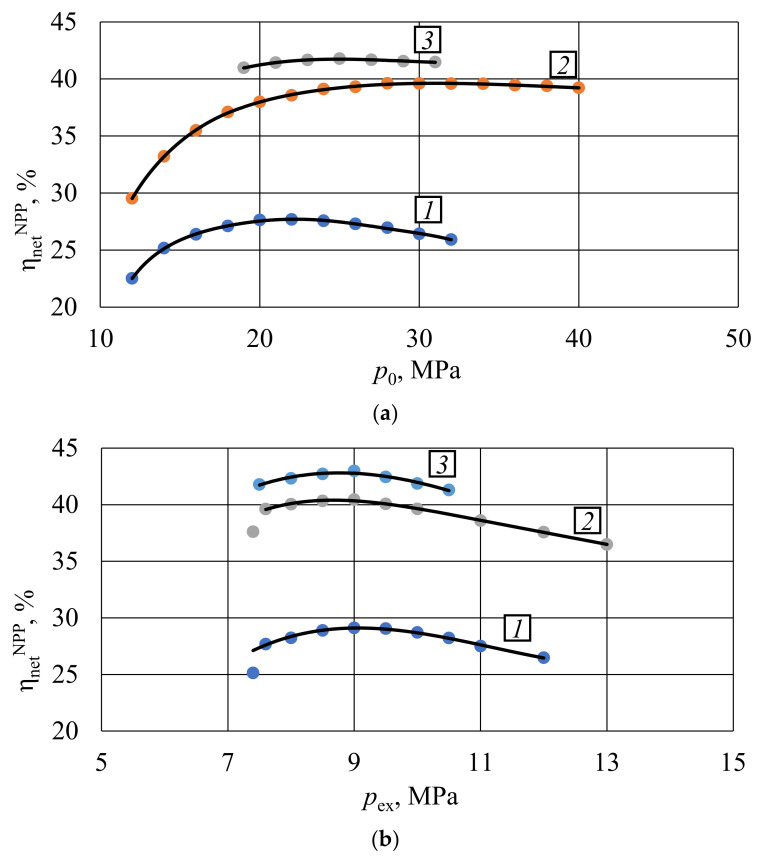
Influence of pressures upon the S–CO_2_ NPP thermal efficiency: (**a**) inlet pressure; (**b**) outlet pressure. Dependencies are given for different reactor types: 1—VVER-1000; 2—BN-800; 3—BREST-OD-300.

Results of the turbine inlet pressure *p*_0_ optimization presented in Figure 4a show that the carbon dioxide NPP net efficiency increase follows the inlet pressure raise in the following pressure ranges:

- From 12 MPa (22.51%) to 22 MPa (27.68%)—for reactor VVER-1000;
- From 12 MPa (29.53%) to 28 MPa (39.61%)—for reactor BN-800;
- From 19 MPa (40.97%) to 25 MPa (41.78%)—for reactor BREST-OD-300.

Reduction of the carbon dioxide NPP thermal efficiency occurs at the inlet pressure increase in the following ranges:

- From 22 MPa (27.68%) to 32 MPa (25.92%)—for reactor VVER-1000;
- From 28 MPa (39.61%) to 40 MPa (39.22%)—for reactor BN-800;
- From 25 MPa (41.78%) to 31 MPa (41.47%)—for reactor BREST-OD-300.

Maximal values of carbon dioxide NPP thermal efficiency occur in the following initial pressure values:

- 22 MPa—for reactor VVER-1000;
- 28 MPa—for reactor BN-800;
- 25 MPa—for reactor BREST-OD-300.

Studies of the turbine outlet pressure p_c_‘s influence upon the carbon dioxide NPP thermal efficiency are carried out at fixed optimal initial pressures. Results in Figure 4b demonstrate equal values of the optimal exhaust pressure 9 MPa for the reviewed facilities. Maximal net efficiency values are 29.12, 40.44, and 42.98% for the recompression cycles with reactors VVER-1000, BN-800, and BREST-OD-300, respectively.

Carbon dioxide cycles have high turbine inlet pressures that are typical for supercritical steam turbines. Nevertheless, the carbon dioxide pressure ratios are 2.5 to 3.0, which is even lower than that in gas turbines. This feature is caused by the requirement of the final pressure above 7.4 MPa. On the other side, the compressor power consumption becomes remarkably high for inlet pressure above 30–35 MPa. Optimal turbine inlet and outlet pressure values are conditioned by the possibility to reach the optimal ratio of the turbomachines′ useful power and the cooler′s losses.

**Figure 5:**
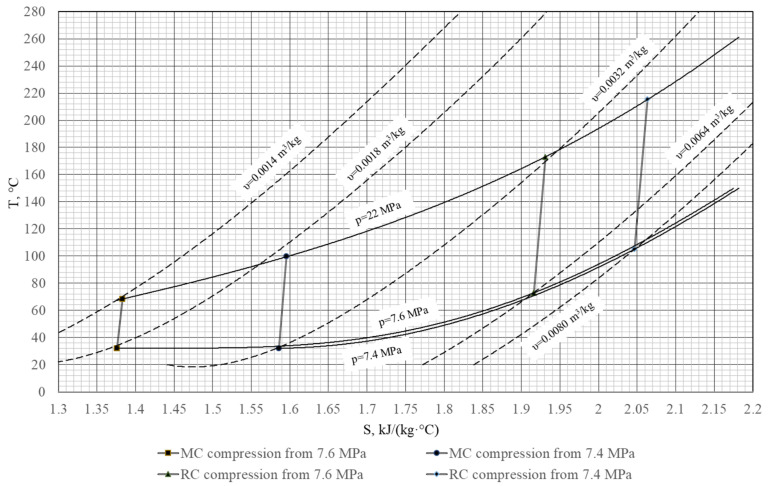
Main compressor and recompressing compression T-S diagram.

When the cycle parameters approach the carbon dioxide subcritical area, the thermal efficiency changes remarkably. The carbon dioxide specific volume grows about 1.8 times (from 0.0018 to 0.0032 m^3^/kg) at a small reduction of final pressure from 7.6 to 7.4 MPa and at 32 °C (Figure 5). At the same time, the compression work in the main compressor grows by 33%, and the compressor outlet temperature grows up to 31.5 °C (Table 6). In these conditions, the thermal efficiency is an order below the studied trend at the turbine outlet pressure of 7.4 MPa.

### 4.2. Thermodynamic Optimization of the Recompression Ratio in the S–CO_2_ NPP

Recompression ratio is an important parameter that considerably influences the cycle′s thermal efficiency. For the turbine inlet and outlet pressure optimization, it is fixed at a 29–30% level. The studies of recompression ratio′s influence upon the net efficiency are carried out at the initial pressure optimal values of 22, 28, and 25 MPa for the VVER-1000, BN-800, and BREST-OD-300 reactors, respectively, and the earlier obtained 9 MPa exhaust pressure.

**Figure 6:**
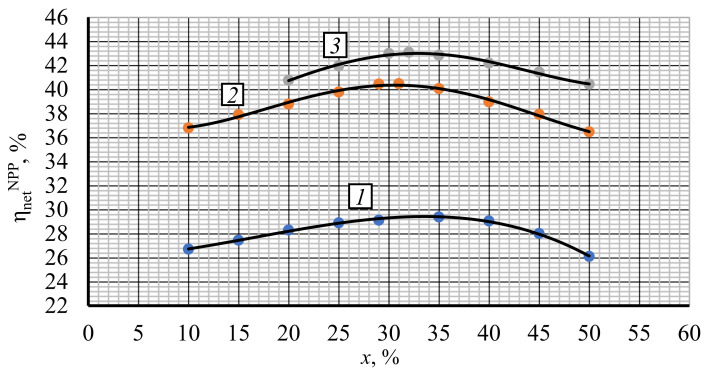
Net efficiency vs. recompression ratio x for different reactor types. 1—VVER-1000; 2—BN-800; 3—BREST-OD-300.

Figure 6 presents the recompression ratio optimization results. The results show the NPP net efficiency increase in the following recompression ratio increase ranges:

- From 10% (26.71%) to 35% (29.39%)—for reactor VVER-1000;
- From 10% (36.80%) to 31% (40.48%)—for reactor BN-800;
- From 20% (40.76%) to 32% (43.08%)—for reactor BREST-OD-300.

On the other side, the NPP net efficiency drops down in the following recompression ratio increase ranges:

- From 35% (29.39%) to 50% (26.10%)—for reactor VVER-1000;
- From 31% (40.48%) to 50% (36.46%)—for reactor BN-800;
- From 32% (43.08%) to 50% (40.42%)—for reactor BREST-OD-300.

Thus, the NPP net efficiency reaches its maximal values at 35, 31, and 32% recompression ratios in VVER-1000, BN-800, and BREST-OD-300 reactors, respectively. Recompression ratios above these values increase the recompression compressor mass flow and its power consumption. On the other side, recompression values below the optimum level increase the cooler heat losses.

Thus, the recompression ratio optimization follows the relation: the higher the cycle′s initial pressure is, the smaller the recompression ratio is. At the turbine inlet pressure increase and the compressor outlet temperature increase, the result is that the recompression compressor inlet temperature and the compression work increase. Because of that, the cycle efficiency may then be improved by an increase of the main compressor load with colder inlet flow. This may be reached by the recompression ratio reduction.

## 5. Discussion

Change of the working fluid from water to carbon dioxide influences the energy equipment composition and performance, as well as the parameters of fluid in the thermal scheme. This approach produces changes in the facility efficiency and the installed capacity price. Finally, it influences the electricity production′s primary cost. The carbon dioxide NPP thermodynamic investigation and development makes a base for the comparison of the equipment composition, the key parameters in the thermal flow schemes, and evaluation of the power production effect caused by the working fluid change.

The cycles′ working fluid parameters are different; therefore, the power facility equipment will be also different, which influences the facility price and the power auxiliary consumption. For example, in the Brayton recompression cycle with VVER-1000, the auxiliary consumption consists of the CO_2_ compressor power, the cooler flow pump circulation power, the main circulation pump in the VVER-1000, and BN-800 reactors that provide the heat carrier circulation in the reactor circuits, and the compressors drive power. In the NPP with water working fluid, auxiliary consumption consists of the condensate circulation, feeding and booster pumps power, and the same main circulation pumps as in the recompression cycle of the VVER-1000 and BN-800 reactors.

**Figure 7:**
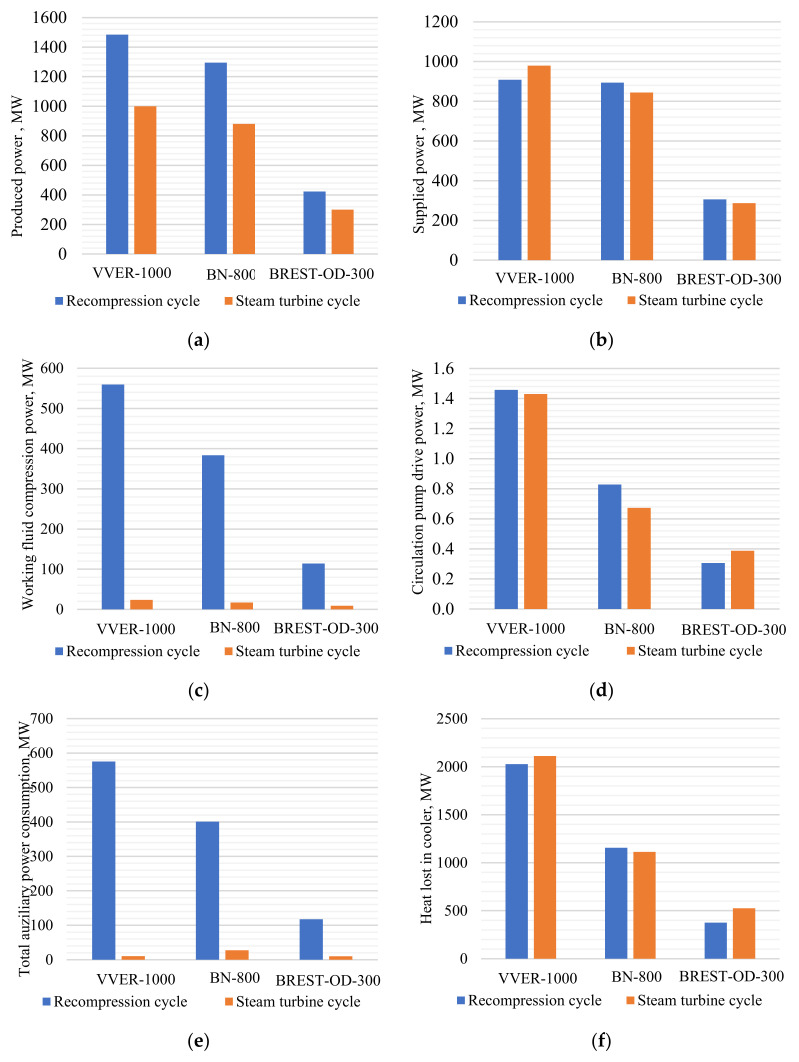
Technical performance comparison of recompression cycle and steam turbine unit for NPP with the different reactor types: (**a**) gross power; (**b**) net power; (**c**) working fluid compression power consumption; (**d**) circulation pumps′ power consumption; (**e**) total auxiliary power consumption; (**f**) cooler heat losses.

Comparing the equipment composition in carbon dioxide NPP and steam turbine NPP, the following performance characteristics may be highlighted:

- Electric power production (Figure 7a);
- Grid supplied electric power (Figure 7b), which is the difference between the power production and the total auxiliary power consumption (Figure 7e);
- The main auxiliary power consumption in the NPP carbon dioxide and steam turbine external circuit, including the compressor power (Figure 7c), the power of the condenser, and cooler circulation pumps (Figure 7d);
- The cold source specific losses (Figure 7f) are the cold source losses divided by the reactor thermal power.

The turbine power output depends upon its heat drop and the working fluid mass flow. A carbon dioxide NPP available heat drop is 12 times smaller than the steam turbine one, but the carbon dioxide turbine mass flow is 12 times larger than the steam turbine one. In the carbon dioxide cycle, there is no regeneration bleeding, so the whole CO_2_ flow produces power in the whole turbine flowpath. Therefore, the carbon dioxide turbine power production is approximately 1.5 times larger (Figure 7a). On the other side, the main carbon dioxide power consumption is the CO_2_ recompression compressor drive power (Figure 7c), which is about 18% of the reactor thermal power.

A steam turbine NPP has much lower auxiliary power consumption than a carbon dioxide one. This is explained as follows. In a steam turbine NPP, the liquid working fluid pressure is raised up to the subcritical pressure, and in the carbon dioxide Brayton cycle, the gas working fluid is compressed up to the supercritical state (Figure 7c).

**Figure 8:**
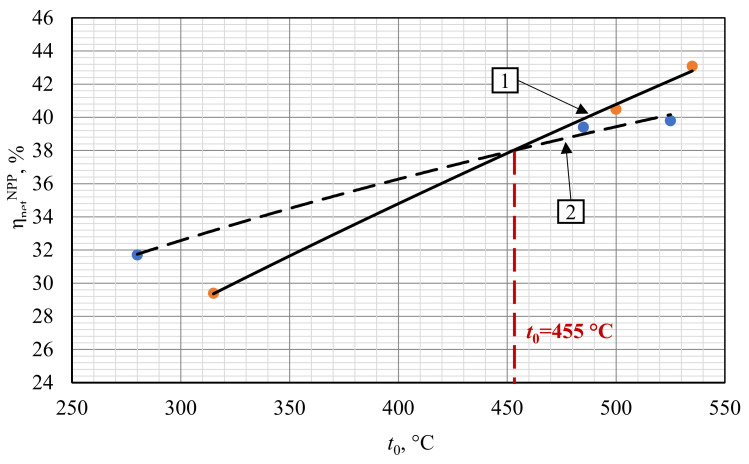
Influence of the working fluid type and initial temperature upon the NPP net efficiency. 1—S–CO_2_ NPP; 2—steam turbine NPP.

A comparison of the water and carbon dioxide heat carrier NPP presented in Figure 8 leads to the following conclusion. Changing a working fluid from steam to carbon dioxide in the VVER-1000 reactor does not increase the power facility efficiency. On the other hand, the BN-800 and BREST-OD-300 efficiency increases. It is worth mentioning that at the turbine inlet temperature t_0_ above 455 °C, it is reasonable to apply the carbon dioxide working fluid due to the higher thermal efficiency. At the initial temperature of 525 °C, the carbon dioxide NPP net efficiency is more than 2% higher than the steam turbine one.

The higher S–CO_2_ NPP net efficiency compared to the steam turbine NPP at the initial temperature above 455 °C may be explained as follows. In the carbon dioxide power cycle, the initial temperature increase is followed by the significant increase of hot source gas inlet temperature (Table 7). (The flow temperature at the inlet of the heat exchanger transfers heat from the NPP internal circuit to the external one.) This increase is due to the carbon dioxide facility′s effective regeneration system that increases the mean integral temperature of the heat supply into the cycle, and the related cycle thermal efficiency. In turn, the initial temperature increase for the traditional steam turbine NPP equipped with BN-800 and BREST-OD-300 reactors is not followed by the feedwater temperature increase because it is limited by the upper bleeding pressure, which is relatively low in the subcritical steam turbine cycles.

Despite the efficiency increase, the conclusion on the reasonability of the change to carbon dioxide working fluid in NPP with BN-800 and BREST-OD-300 reactors may be issued only based on the technical and financial performance of the traditional and prospective facilities. Therefore, there is a need for development of the new large power carbon dioxide operating equipment and assessment of its mass and dimensions. The turbine and equipment buildup investments will be remarkably different. Thus, in further investigation, it is important to develop a scientific approach to turbine and heat exchanger equipment design for the carbon dioxide NPP.

## 6. Conclusions

(1) The published literature review shows that the most prospective S–CO2 power cycle is the Brayton cycle with recompression. The facilities operating this cycle have a remarkable degree of flue gas heat utilization and low cold source losses. This is due to the split of compressed flow into two parts and by the introduction of low-temperature and high-temperature regenerative heat exchangers. This cycle′s thermal efficiency may be above 40%, which determines its relevance for application for the NPP equipped with the existing reactors including VVER-1000, BN-800, and BREST-OD-300.
(2) The computer simulation models of the S–CO2 NPP with VVER-1000, BN-800, and BREST-OD-300 were developed to evaluate the key thermodynamic parameters across the thermal flow scheme and thermal efficiency. These are used for the thermodynamic optimization.
(3) The 1 MPa turbine inlet pressure increase causes the following carbon dioxide NPP net efficiency increase:
  - 0.52% in facilities with VVER-1000 reactor in the initial pressure range 12–22 MPa;
  - 0.63% in facilities with BN-800 reactor in the initial pressure range 12–28 MPa;
  - 0.14% in facilities with BREST-OD-300 reactor in the initial pressure range 19–25 MPa.
  In turn, the 1 MPa turbine inlet pressure increase causes the following carbon dioxide NPP net efficiency reduction:
  - 0.18% in facilities with VVER-1000 reactor in the initial pressure range 22–32 MPa;
  - 0.03% in facilities with BN-800 reactor in the initial pressure range 28–40 MPa;
  - 0.05% in facilities with BREST-OD-300 reactor in the initial pressure range 25–31 MPa.
(4) The 0.1 MPa turbine outlet pressure reduction is causing the following mean carbon dioxide NPP net efficiency reduction:
  - 0.1% in facilities with VVER-1000 reactor in the range 9–12 MPa and BREST-OD-300 reactor in the range 9–13 MPa;
  - 0.08% in facilities with BN-800 reactor in the range 9–10.5 MPa.
  In turn, every 0.1 MPa turbine outlet pressure reduction in the range below 9 MPa is followed by a 0.1% net efficiency reduction.
(5) The mean thermal efficiency increase at a 1% recompression ratio increase is:
  - 0.1% in facilities with VVER-100 reactor at the recompression ratio range 10–35%;
  - 0.18% in facilities with BN-800 at the recompression ratio range 10–31%;
  - 0.19% in facilities with BREST-OD-300 at the recompression ratio range 20–32%.
  In turn, the mean thermal efficiency reduction at a 1% recompression ratio increase is:
  - 0.2% in facilities with VVER-1000 reactor at the recompression ratio range 35–50%;
  - 0.21% in facilities with BN-800 reactor at the recompression ratio range 31–50%;
  - 0.15% in facilities with BREST-OD-300 reactor at the recompression ratio range 32–50%.
(6) The results of comparison of the NPP power production performance working with water and carbon dioxide working fluids show that the change of steam to carbon dioxide is reasonable at the initial working fluid temperature above 455 °C. This maximal thermal efficiency increase is due to the more effective regeneration system operation.

## Figures and Tables

**Table 1:** Thermophysical properties of different working fluids at typical parameters for steam, gas, and carbon dioxide turbine facilities.

Facility Type	Turbine Inlet/Outlet Temperature and Pressure	Turbine Inlet/Outlet Working Fluid Density, kg/m^3^	Turbine Inlet/Outlet Working Fluid Kinematic Viscosity, 10^7^ m^2^/s
Steam turbine power plant (H_2_O)	540 °C, 23.5 MPa/29 °C, 4 kPa	74.6/0.004	4.3/26.5
Combined cycle power plant (air)	1100 °C, 1.3 MPa/515 °C, 0.1 MPa	3.3/0.44	162.4/840.4
S–CO_2_ power plant (CO_2_)	540 °C, 25 MPa/407 °C, 8 MPa	155.8/70.8	2.4/4.5

**Table 2:** An influence of parameters on the S–CO_2_ recompression Brayton cycle efficiency.

Parameters	Parameter Range	Influence upon Efficiency	Reference
Compressor outlet pressure, *p*_max_, MPa	15–30	1 MPa increase ⇒ 0.2–0.8% efficiency increase	[7]
Turbine inlet temperature, *t*_0_, °C	550–850	100 °C increase ⇒ 4% efficiency increase	[7]
Turbine inlet temperature, *t*_0_, °C	500–1000	100 °C increase ⇒ 3.33% efficiency increase	[6]
Turbine inlet temperature, *t*_0_, °C	500–1000	100 °C increase ⇒ 2% efficiency increase	[11]
Turbine inlet temperature, *t*_0_, °C	550–700	100 °C increase ⇒ 5% efficiency increase	[13]
Turbine inlet temperature, *t*_0_, °C	32–50	10 °C increase ⇒ 2,7% efficiency reduction	[7]
Turbine inlet pressure, *p*_0_, MPa	10–25	1 MPa increase ⇒ 0.2–0.4% efficiency increase	[14]
Turbine efficiency, η, %	85–95	5% increase ⇒ 2% efficiency increase	[13]
Compressor efficiency, η, %	85–90	5% increase ⇒ 1% efficiency increase	[13]
Regenerators minimal temperature difference, Δ, °C	5–10	5 °C increase ⇒ 1% efficiency reduction	[13]

**Table 3:** Thermodynamic optimization results for the S–CO_2_ Brayton cycles.

	With Regeneration	With Reheat	With Inter-Cooling	With Partial Cooling	With Recompression
Cycle initial temperature *t*_0_, °C	550	550	550	550	550
Cycle initial pressure, *p*_0_, MPa	35	35	45	25	35
Cycle final pressure, *p*_c_ MPa	7.5	7.5	7.5	5	7.7
Reheat pressure, *p*_rh_, MPa	–	10	–	–	–
Heat supply to the cycle in reactor, *Q*_0_, MW	33.03	33.91	39.12	28.85	26.43
Heat loss from the cycle in coolers, *Q*_c_, MW	19.09	19.09	22.17	15.84	13.88
Turbine power *N*_t_, MW	19.66	20.54	22.46	20.28	18.96
Compressor power, *N*_c_, MW	5.73	5.73	6.05	7.35	6.46
High-temperature and low-temperature heat exchanger thermal power, *Q*_reg_, MW	30.80	44.92	32.60	18.61/9.73	13.00/20.22
Working fluid temperature at the source of heat inlet, *t*_react.in_, °C	295	427/398	280	320	344
Cycle net efficiency, η, %	42.2	43.70	41.96	44.80	47.28

**Table 4:** Existing NPP internal circuit nominal performance.

	Reactor Type		
	VVER-1000	BN-800	BREST-OD-300
Reactor thermal power, MWt	3000	2100	700
First cicuit heat carrier mass flow, kg/s	17,778 (H_2_O)	8550 (Na)	41,600 (Pb)
First cicuit heat carrier pressure, MPa	15.7	0.16	0.155
First cicuit inlet temperature, °C	289	354	420
First cicuit outlet temperature, °C	322	547	535
Second circuit heat carrier mass flow, kg/s	1633 (H_2_O)	8418 (Na)	420 (H_2_O)
Second circuit heat carrier pressure, MPa	5.9	1.96	17
Second circuit heat carrier inlet temperature, °C	220	309	340
Second circuit heat carrier outlet temperature, °C	274	505	505
Third circuit heat carrier mass flow, kg/s	–	875 (H_2_O)	–
Third circuit heat carrier pressure, MPa	–	14.2	–
Third circuit heat carrier inlet temperature, °C	–	210	–
Third circuit heat carrier outlet temperature, °C	–	485	–
First circuit main circulation pump power, MW	5.3	5	–
Second circuit main circulation pump power, MW	–	2.5	–

**Table 5:** Initial data for the modeling of the S–CO_2_ nuclear power plants.

	Reactor Type		
	VVER-1000	BN-800	BREST-OD-300
Basepoint values of optimization variables			
Turbine inlet pressure, MPa	20		
Turbine outlet pressure, MPa	7.6		
Cycle recompression rate, %	29		
All heat flow versions fixed parameters			
Cooler minimal temperature difference, °C		17	
Working fluid temperature at the main compressor inlet, or the cycle minimal temperature, °C		32	
Cooler circulation water pressure, bar		1.3	
Cooler inlet cooling water temperature, °C		15	
Cooling water temperature at the cooler outlet, °C		25	
Heat exchangers temperature difference, °C		5	
Main compressor specific internal efficiency, %		90	
Recompressing compressor specific internal efficiency, %		90	
Turbine specific internal efficiency, %		90	
Pumps specific internal efficiency, %		75	
Power generator/motor efficiency, %		99	
Mechanical efficiency, %		99	
Heat exchanger from reactor efficiency, %		98	
Heat transportation efficiency, %		99	

**Table 6:** Initial pressure influence on the main thermodynamic parameters of working fluid in compressors.

Element	Process Station	Value			
		*T*, °C	*p*, MPa	*S*, KJ/(kg·°C)	υ, m^3^/kg
Main compressor, compression from 7.4 MPa	before compression	32	7.4	1.5857	0.00317
	after compression	99.81	22	1.5956	0.00189
Main compressor, compression from 7.6 MPa	before compression	32	7.6	1.3759	0.00179
	after compression	68.32	22	1.3831	0.00142
Recompressing compressor, compression from 7.4 MPa	before compression	105	7.4	2.0463	0.008
	after compression	215.63	22	2.0633	0.00371
Recompressing compressor, compression from 7.6 MPa	before compression	73	7.6	1.916	0.00636
	after compression	172.95	22	1.931	0.00311

**Table 7:** Temperature comparison at the hot source inlet for the NPP with water and carbon dioxide working fluids.

Reactor	Working Fluid Temperature at the Hot Source Inlet, °C	
	S–CO_2_ Power Cycle (CO_2_)	Steam Turbine Power Cycle (Feed Water)
VVER-1000	198	220
BN-800	328	210
BREST-OD-300	367	–

## Data Availability

Not applicable.
